# Probing the structure–function relationship with neural networks constructed by solving a system of linear equations

**DOI:** 10.1038/s41598-021-82964-0

**Published:** 2021-02-15

**Authors:** Camilo J. Mininni, B. Silvano Zanutto

**Affiliations:** 1 Consejo Nacional de Investigaciones Científicas y Técnicas, Instituto de Biología y Medicina Experimental, Buenos Aires, Argentina; 2 Universidad de Buenos Aires, Facultad de Ingeniería, Instituto de Ingeniería Biomédica, Buenos Aires, Argentina

**Keywords:** Dynamical systems, Network models

## Abstract

Neural network models are an invaluable tool to understand brain function since they allow us to connect the cellular and circuit levels with behaviour. Neural networks usually comprise a huge number of parameters, which must be chosen carefully such that networks reproduce anatomical, behavioural, and neurophysiological data. These parameters are usually fitted with off-the-shelf optimization algorithms that iteratively change network parameters and simulate the network to evaluate its performance and improve fitting. Here we propose to invert the fitting process by proceeding from the network dynamics towards network parameters. Firing state transitions are chosen according to the transition graph associated with the solution of a task. Then, a system of linear equations is constructed from the network firing states and membrane potentials, in a way that guarantees the consistency of the system. This allows us to uncouple the dynamical features of the model, like its neurons firing rate and correlation, from the structural features, and the task-solving algorithm implemented by the network. We employed our method to probe the structure–function relationship in a sequence memory task. The networks obtained showed connectivity and firing statistics that recapitulated experimental observations. We argue that the proposed method is a complementary and needed alternative to the way neural networks are constructed to model brain function.

## Introduction

Understanding brain function requires the construction of models that explain experimental data, which encompass behavioural outcome, anatomical features, neurons biophysics, and coding properties, among others^1,2^. Many kinds of models have been proposed along history. Among them, neural network models are well poised to connect all levels of analysis, from the behavioural to the molecular level, being a natural choice as neurons are the functional units of the brain. Yet, constructing suitable neural network models is not an easy task. Their parameters can be defined according to experimental data, or randomly chosen when the data is not available. However, this approach may fall short given the complexity of the nervous systems. To tackle this issue, theorists have employed optimization algorithms that adjust network parameters in a direction that minimizes a loss function. Conversely, the loss function is constructed in such a way that it is minimized when the network satisfies the desired experimental observations that we are seeking to explain, like an animal’s performance in one or several tasks^3^, connectivity constraints such as Dale’s principle^4^, or connectivity with a certain degree of sparseness^5^. Optimization methods are widely used in artificial intelligence (AI), and the ongoing deep learning revolution has prompted an explosion of fitting algorithms, and the eagerness to take advantage of them to build models of brain function^6,7^. However, AI needs are different from the theoretical neuroscience needs. Artificial intelligence deals with the construction of systems capable of solving difficult tasks, employing very general optimization algorithms for parameter fitting^8^. On the other hand, models in neuroscience are expected to explain how animals behave in simple tasks, yet with biologically plausible neural networks. Simple tasks are desired because behavioural outcomes are easier to interpret, and mechanistic explanations easier to envisage. Thus, in AI the difficulty resides in the task, while in theoretical neuroscience it lies in the restrictions in network design that are imposed by biology. Therefore, methods for parameter fitting in theoretical neuroscience can take advantage of this point – the simplicity of the task – to solve problems that could be too hard to solve with generic optimization algorithms.

One approach that has been overlooked consists in finding the synaptic weights of a network as the solution of a system of equations. For many commonly employed neural network models, neurons perform a weighted sum of their inputs, followed by a non-linear transformation. For this kind of model, the synaptic weights can readily be found by solving a linear system of equations in which the neurons’ firings constitute the coefficient matrix, and the added postsynaptic potentials are the dependent variables. Thus, the problem of finding the network parameters translates into finding a suitable *dynamic* that the network should follow when solving a behavioural task of interest. Although this problem might seem as hard as the former, we show in this work that viable network dynamics can easily be found from the graph of transitions associated with the solution of the task. By doing so, we were able to construct networks with millions of parameters extremely fast, without inefficient searches in parameter space. Moreover, optimization algorithms may have biases for a subset of all possible solutions^9^. These biases depend on the algorithms employed, the hyperparameters, and the regularizations, and the relation between biases and their causes might be difficult to understand or control^10^. In contrast, our method takes samples from a distribution of networks with a desired dynamic, while structural constraints can be imposed in a subsequent step. Since the algorithm proceeds from the network firing states to the network parameters, we call it the *Firing to Parameter* (FTP) algorithm.

The results are organized as follows: first, we describe the key aspects of the FTP algorithm. Second, we employ FTP to construct networks of binary neurons that solve a sequence memory task. We then show how to use the algorithm to obtain networks of desired firing rate and pairwise correlation, and how to incorporate structural constraints, such as Dale’s principle and sparse connectivity. We finally demonstrate how the FTP can be exploited to construct specific null models to test hypotheses regarding the relationship between network structure and function. The networks obtained displayed firing and connectivity features that resemble those observed in neurophysiological experiments.

## Results

### Constructing neural networks with predefined dynamics

**Figure 1:**
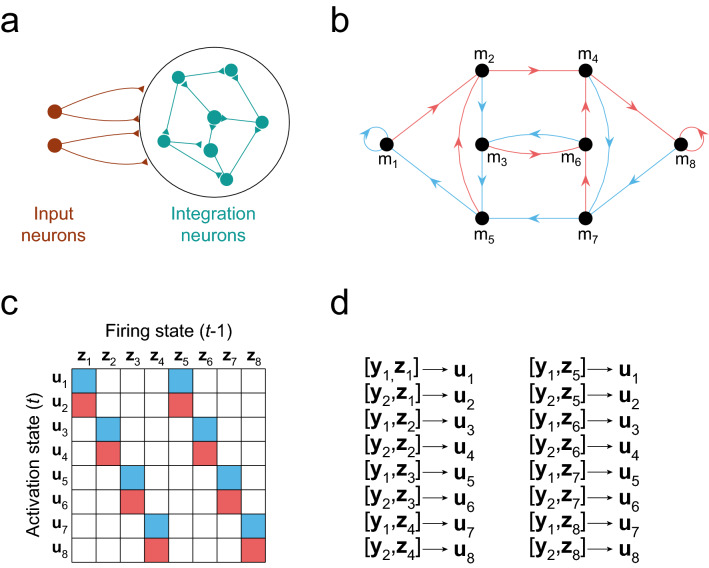
Constructing recurrent networks that follow a predefined transition graph. (**a**) Networks are composed of binary neurons. Input neurons codify stimuli and project to the integration neurons through synaptic weights ${\mathbf{W}}^{in}$. Integration neurons are recurrently connected through synaptic weights ${\mathbf{W}}^{rec}$. (**b**) Transition graph showing transitions between network states during execution of the s-task, for $\tau = 3$. Each node in the graph is a network state, and the directed edges depict transitions between states after stimuli presentation (blue for $s_{1}$, red for $s_{2}$). Each possible sequence of 3 stimuli is codified by exactly one network state. Nodes are numbered such that transitions can be represented in a simple transition matrix. (**c**) Transition matrix associated with the transition graph in panel (**b**). It shows the activation states **u** that are reached when integration neurons are in a population firing state **z**, and $s_{1}$ (blue) or $s_{2}$ (red) are presented. (**d**) Same transitions depicted in panels (**b**) and (**c**), but explicitly showing vectors ${\mathbf{u}}_{i}$ and vectors ${\mathbf{c}}_{i}$, which are the concatenation of one **y** and one **z**. The index *i* is such that **z**_i_ and **u**_*i*_ are the firing state and activation vectors corresponding to network state *m*_*i*_.

We will consider a network of *N*_*rec*_ recurrently connected binary neurons (the *integration* neurons), which receive information about the environment from a set of *N*_*in*_ input neurons (Fig. 1a). The temporal evolution of the network is dictated by the standard equations of the linear-threshold neuron model:

1 $${\mathbf{u}}(t) = {\mathbf{y}}(t){\mathbf{W}}^{in} + {\mathbf{z}}(t - 1){\mathbf{W}}^{rec}$$

2 $${\mathbf{z}}(t) = {\text{H}} \left( {{\mathbf{u}}(t) - {{\varvec{\uptheta}}}} \right)$$

where **W**^*in*^ and **W**^*rec*^ are synaptic weights matrices of input and integration neurons, vector **y** contains firing states of input neurons, which codify the stimuli presented, ${{\varvec{\uptheta}}}$ is a vector of neuron thresholds, and H stands for the Heaviside function. Vector **u** is a real-valued vector of length *N*_*rec*_ that collects the *network activation states*, akin to membrane potentials, and **z** is a vector of zeros and ones that collects the *network firing states*. At any given time *t* the recurrent network can adopt one out of *M* network firing states *m*, defined by vector **z**_*m*_.

Equation (1) shows how firing states and activation states are linearly related through the synaptic weights. Thus, the weight matrices can be computed exactly by solving a linear system of equations, provided that firing states and the resulting activations are known. To specify, we define ${\mathbf{c}}_{s,m} = [{\mathbf{y}}_{s} \,\,\,{\mathbf{z}}_{m} ]$, the concatenation of the input firing state during presentation of stimulus *s*, and the network firing state when the network is at state *m*. Next, we construct a matrix **C** whose rows are vectors ${\mathbf{c}}_{s,m}$ for all the combinations of stimuli and network states we want to include in the proposed dynamic. Then, the following system of linear equations follows:

3 $${\mathbf{CW}} = {\mathbf{U}}$$

where matrix ${\mathbf{W}} = [{\mathbf{W}}^{in} \,\,\,{\mathbf{W}}^{rec} ]$ is the concatenation of the input and recurrent synaptic weights. Here the $i^{th}$ row in **U** is the activation state ${\mathbf{u}}(t)$ when the $i^{th}$ row of **C** is $[{\mathbf{y}}(t)\,\,{\mathbf{z}}(t - 1)]$. Then, **W** can be computed as:

4 $${\mathbf{W}} = {\mathbf{C}}^{ + } {\mathbf{U}}$$

where **C**^+^ stands for the pseudoinverse of **C**. We employed the pseudoinverse because it gives the solution that minimizes the Frobenius norm^11^. In this manner, synaptic weights are going to be small in absolute value, which is desirable since biological synapses are constrained in the number of receptors and vesicles.

Matrices **C** and **U** should be picked in such a way that they instantiate the state transitions exhibited by a network while solving a target task, e.g., transitions to different network states after presentation of different stimuli in a discrimination task. Simple tasks like the ones employed in behavioural neuroscience exhibit simple state transitions, hence the transition graph is known. Therefore, it only remains to find the actual vectors **u** and **c**. A naïve approximation to this problem would be to pick vectors **u** at random, apply the thresholds to obtain the associated vectors ${\mathbf{z}}$, and construct matrices **C** and **U** by following the desired transition graph. However, by doing so we likely end up having an inconsistent system of equations, meaning that no network of neurons can follow those state transitions. To understand this important point, we may consider the case of two stimuli *s*_1_ and *s*_2_, codified by input neurons with firing states **y**_1_ and **y**_2_ respectively. Then, each vector $[{\mathbf{y}}_{1} \,\,\,{\mathbf{z}}_{m} ]$ can be expressed as a linear combination of $[{\mathbf{y}}_{2} \,\,\,{\mathbf{z}}_{m} ]$ and vectors $[{\mathbf{y}}_{1} \,\,\,{\mathbf{z}}_{P} ]$ and $[{\mathbf{y}}_{2} \,\,\,{\mathbf{z}}_{P} ]$, where vector ${\mathbf{z}}_{P}$ can be any vector taken from the set of all firing states the network can adopt:

5 $$[{\mathbf{y}}_{2} \,\,\,{\mathbf{z}}_{m} ] = [{\mathbf{y}}_{1} \,\,\,{\mathbf{z}}_{m} ] - [{\mathbf{y}}_{1} \,\,\,{\mathbf{z}}_{P} ] + [{\mathbf{y}}_{2} \,\,\,{\mathbf{z}}_{P} ]$$

Thus, ${\text{rank}} ({\mathbf{C}}) = M + 1$. Following the Rouché-Capelli theorem^12^, Eq. (3) has a solution if and only if ${\text{rank}} ({\mathbf{C}}) = {\text{rank}} ([{\mathbf{C}}\,\,\,{\mathbf{U}}])$, being $[{\mathbf{C}}\,\,\,{\mathbf{U}}]$ the augmented matrix. Yet, if we choose the rows of **U** randomly, when adjoined to matrix **C** the linear dependencies expressed in Eq. (5) will be broken, and the resulting augmented matrix will have rank above *M* + 1. However, if the rows of **U** are linearly combined following the linear combinations present in **C**, the resulting system of equations will be consistent, and its solution will retrieve matrix **W**. This is the key aspect of the FTP algorithm, which can be succinctly stated as:

1. Find the transition graph between network states that solve the target task.
2. Choose activation states **u** from a desired distribution.
3. Use vectors **u** and associated vectors **c** to construct matrices **U** and **C** following the transition graph.
4. Linearly combine rows in **U** to preserve the linear combinations among rows of **C** (Eq. 5)

### Testing the FTP algorithm in a sequence memory task

In the following we exemplify the utility of the FTP algorithm by constructing networks that solve a sequence memory task (s-task). In this task two stimuli *s*_1_ and *s*_2_, codified by input neurons firing states **y**_1_ and **y**_2_, are sequentially presented at each time step, chosen randomly with equal probability. To obtain reward at time step *t* the network has to recall the stimulus presented at time step $t - \Delta t$. By recall we mean that the stimulus at time $t - \Delta t$ is univocally codified by the network firing state at time *t*. Consequently, successful behaviour requires to have a memory of stimuli sequences of length $\tau = \Delta t + 1$. We chose this task because its complexity grows exponentially with $\tau$, making it a good benchmark to test the computational efficiency of the algorithm. It has also been shown that neural populations are sensitive to stimulus presentation ordering, and the coding of such sequences is incompletely understood^13^. Thus, we decided to exploit the FTP capabilities to study the neural dynamics and connectivity in networks of binary neurons that are capable of solving the s-task. Figure 1b shows the network states and state transitions gated by the stimuli when solving the s-task with $\tau = 3$. Despite its complexity, the state transitions required to solve the task have a stereotyped structure, which is evident when network states are numbered properly (Fig. 1b–d). The transition graph in Fig. 1b shows the state transitions any network that solves the s-task should follow. With the transition matrix structure at hand, we can construct matrices **C** and **U** (see details in the “Methods” section).

**Figure 2:**
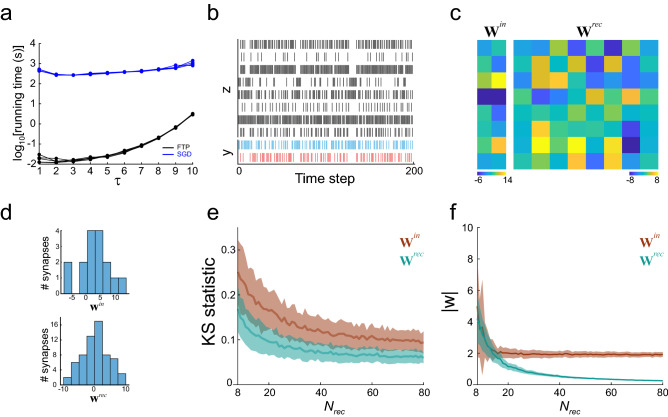
Solving the s-task with the FTP algorithm. (**a**) Efficiency of FTP and SGD, measured as the time expended in finding a solution network for s-tasks of different $\tau$. The time expended by the FTP algorithm is orders of magnitude lower than the time expended by the SGD algorithm (n = 5 networks for each $\tau$ value). (**b**) Raster plot showing the neurons firing states in a network constructed to solve the s-task for $\tau = 3$. The network is composed of 8 integration neurons and 2 input neurons. Each possible sequence of 3 stimuli has a unique network firing state that codifies the sequence. Therefore, the network has 8 possible firing states. (**c**) Input and recurrent synaptic weights of the network. (**d**) Distribution of synaptic weights for input (upper panel) and recurrent (lower panel) synaptic weights, for the same network as in (**b,c**). (**e**) Kolmogorov–Smirnov statistic between the distribution of synaptic weights and a normal distribution of the same mean and variance. As the network size increases, the distribution of synaptic weights gets closer to a normal. Integration neurons weights are closer to a normal distribution than sensory neurons weights. Mean ± SD are shown for n = 100 networks that solve the s-task, with $\tau = 3$. (**f**) Absolute synaptic weight values as a function of network size. Absolute values are higher and of larger variability when the neuron count is close to the number of coded stimuli sequences. As the network size increases the absolute mean value and dispersion decrease. Input neurons weights quickly reach a minimum, while integration neurons weights decrease in the entire range of network sizes. Mean ± SD are shown for n = 100 networks that solve the s-task, with $\tau = 3$.

We assessed the performance of the algorithm by measuring the time it takes to find networks that solve the s-task ($N_{rec} = 1024$, $\tau = 1$ to $\tau = 10$) and comparing those times with the time taken by a stochastic gradient descent (SGD) algorithm (*Adam* optimizer^14^, adapted to fit threshold units^15^). The FTP algorithm outperformed SGD by at least two orders of magnitude (Fig. 2a). This is not surprising when considering that a linear system of equations can be solved in polynomial time, while SGD (and any other fitting algorithm) requires huge numbers of network evaluations to obtain a single set of parameters updates. An example network constructed with FTP is shown in Fig. 2b,c, of $N_{rec} = 8$ neurons and capable of solving an s-task with $\tau = 3$. The resulting synaptic weight distribution had zero mean and resembled a normal distribution, at least for the **W**^*rec*^ values (Fig. 2d). In fact, the synaptic weight distributions became progressively closer to a normal distribution as more neurons were employed in network construction (Fig. 2e). We also noted that the absolute weight value decreased, especially for **W**^*rec*^ values (Fig. 2f), which can be explained by thinking that more neurons imply more parameters and hence more degrees of freedom to reach a lower Frobenius norm. This observation will become important later when imposing structural constraints to the network.

### Specifying the firing rate and pairwise correlation of the network

**Figure 3:**
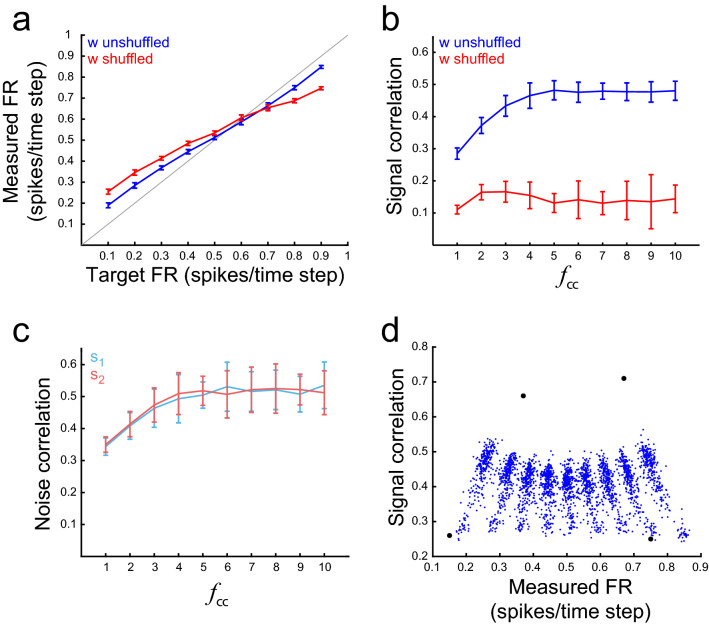
Imposing activity constraints with FTP. (**a**) FR measured in networks constructed to solve the s-task, as a function of the target FR. The FR of networks constructed with the FTP algorithm is close to the target FR (blue line). There is a tendency to obtain lower firing rates for target FR values above 0.5 spikes/time step, and higher firing rates for target FR values below 0.5 spikes/time step (the gray line is the identity function). The same networks with their synaptic weights shuffled (red line) show a similar relationship between target FR and measured FR, albeit with a lower slope. A total of 30 networks were generated for each target FR. Weights in ${\mathbf{W}}^{in}$ and ${\mathbf{W}}^{rec}$ were shuffled separately. Mean ± SD are shown. (**b**) Correlation between pairs of integration neurons as a function of the scaling factor *f*_*cc*_. Pairwise correlation, computed over all time steps, increases with *f*_*cc*_ until it saturates at CC = 0.48 for $f_{cc} \ge 5$ (blue line). Networks with their afferent synaptic weights shuffled (red line) show low correlation, invariant to *f*_*cc*_. A total of 30 networks were constructed for each *f*_*cc*_ value, with target FR set to 0.1 spikes/time step. Mean ± SD are shown. (**c**) Pairwise correlation computed separately for $s_{1}$ and $s_{2}$ (noise correlation). The correlation coefficient increases with *f*_*cc*_, similarly for both stimuli and closely following correlation values in (b). Mean ± SD are shown, n = 30. (**d**) Measured FR as a function of pairwise correlation. Each blue dot shows the FR and CC of one network constructed to solve the s-task with desired FR and CC imposed through ${\mathbf{U}}_{base}$ initialization. Values for 2700 networks are shown. Points form stripes pointing towards FR = 0.5 spikes/time step, each stripe corresponding to networks with the same target FR. As correlation increases, the measured FR tends to 0.5 spikes/time step. Black dots show FR and CC of 4 networks for which desired FR and CC were imposed by evolution of a population of ${\mathbf{U}}_{base}$ matrices.

We want to construct neural network models that not only solve relevant tasks but do so under desired firing constraints, as measured in real brains. Some of these constraints are low firing rates (FR)^16,17^, or low correlation coefficients (CC)^18^. In regular optimization algorithms, these constraints can be imposed by introducing regularization terms in the loss function^5^. On the other hand, in the FTP algorithm the activation states of the network are the result of linearly combining the rows of an initial matrix **U**_*base*_ (see “Methods”). Hence, we can apply firing constraints by appropriately choosing this initial matrix. For example, to attain networks that solve the s-task with low/high firing, it suffices to choose an initial matrix **U**_*base*_ such that, after thresholding, the resulting matrix **C** has few/many ones. Following this procedure, we constructed networks with average FR within a wide range of target FR (Fig. 3a, blue line). Shuffling values within **W**^*in*^ matrices, or within **W**^*rec*^ matrices, produced only small changes to the average FRs (Fig. 3a, red line). This suggests that it is the distribution of synaptic weights the critical statistic that defines the networks average FR, and not the detailed arrangement of these weights in the synaptic weight matrix.

On the other hand, we can construct networks with desired signal correlation, by multiplying ${{\varvec{\Delta}}}$ (the difference in effects produced by *s*_1_ and *s*_2_) by a factor *f*_*cc*_ (Fig. 3b). For networks shown in Fig. 3 ($\tau = 4,f_{r} = 3$), correlations could be modulated in a range between 0.1 and 0.5. Scaling ${{\varvec{\Delta}}}$ was expected to induce signal correlation. However, it also induced noise correlation as a by-product (Fig. 3c). Correlations in networks solving the s-task were significantly higher than correlations in their synaptic weight-shuffled counterparts (Fig. 3b, blue vs. red), which suggests that signal correlations depend on the precise arrangement of values in the weight matrix, and not only on the weight distribution, as was the case with FR. It also suggests that the set of networks that solve the s-task necessarily exhibit correlations above a minimum. On the other hand, correlations did not exceed a certain value: higher correlations may imply a reduced number of network states, incompatible with the number of sequences required to be codified.

Hand-based manipulation of **U**_*base*_ allows us to generate solution networks in a wide range of FR and CC (Fig. 3d, blue dots). An even better control of firing and correlation can be achieved by fitting **U**_*base*_ thought an optimization algorithm. We employed a genetic algorithm (GA) in which the fitness of **U**_*base*_ is a function of the FR and CC computed from the network firing states generated from that **U**_*base*_. Fitting **U**_*base*_ gave access to more extreme values of FR and CC (Fig. 3d, black dots), and kept computational cost low by computing FR and CC from the set of network firing vectors **c** instead of computing the firings by simulating the network. Altogether, both methods (**U**_*base*_ manipulation, or its evolution with a GA) generated networks with desired activity constraints that perfectly solved the task.

### Applying structural constraints with projected gradient descend in isofunction weight space

**Figure 4:**
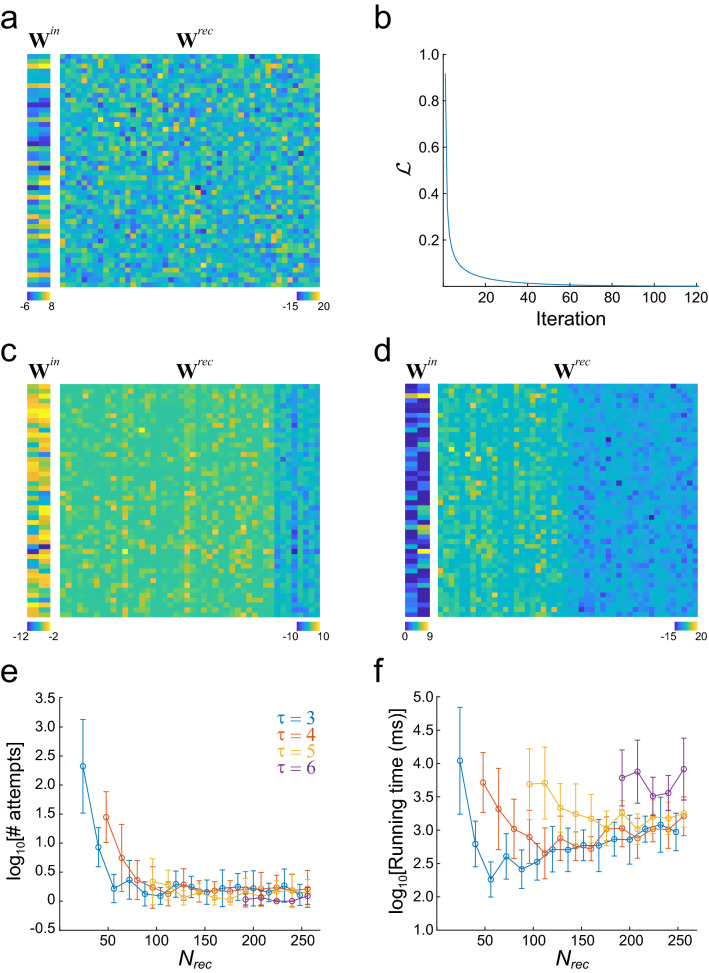
Imposing structural constraints with FTP. (**a**) Synaptic weight matrices ${\mathbf{W}}^{in}$ and ${\mathbf{W}}^{rec}$ of the network obtained through FTP before structural constraints were imposed. The network was constructed to solve the s-task for $\tau = 4$ and $f_{r} = 3$, with a target FR of 0.1 spikes/time step. (**b**) Loss function ${\mathcal{L}}$ as a function of the number of iterations of the PGD algorithm. The loss function falls below the criterium $e_{1} = 10^{ - 3}$ at iteration 121. (**c,d**) Synaptic weight matrices ${\mathbf{W}}^{in}$ and ${\mathbf{W}}^{rec}$ for a network with the same stimulus–response mapping but after applying structural constraints: (**c**) no self-connections, Dale’s principle, with 40 excitatory and 10 inhibitory neurons, and sparsity $sp = 40\%$; (**d**) no self-connections, Dale’s principle, with 26 excitatory and 24 inhibitory neurons, and sparsity $sp = 23\%$. (**e**) Average number of attempts to obtain one network with successful structural fitting, as a function of the number of integration neurons, and for different $\tau$. The number of attempts is high when the neuron number is low, but it decreases fast as the neuron number increases. From 60 neurons onwards, less than five attempts are needed, on average, to obtain one network with the desired structural constraints. Mean ± SD are shown. (**f**), total running time to obtain one network with successful structural fitting, as a function of the number of integration neurons, and for different $\tau$ (color code as in (**e**)). Running time decreases and then increases for $\tau = 3$ and $\tau = 4$. The case of $\tau = 6$ is the one with more neurons and equations to solve and present some of the highest running times, even when the number of neurons is high. Nevertheless, all average running times are in the order of tens of seconds.

Networks generated so far share one structural constraint: their synaptic weight matrix is the one that minimizes the Frobenius norm. Other relevant structural constraints, such as the low probability of self-connections^19^, Dale’s principle^4^, or sparse connectivity are not satisfied. Since these structural constraints are key experimentally observed features, we were interested in imposing such constraints onto the **W** obtained by the algorithm. To do this we followed a projected gradient descent (PGD) approach^20^, taking advantage of the fact that the loss function ${\mathcal{L}}$, which encloses the structural constraints, is a linear function with respect to the synaptic weights, and that the matrix **W** can be changed without changing the stimulus–response mapping (see “Methods”). To exemplify the procedure, we constructed a network that solves the s-task for $\tau = 4$, with $f_{r} = 3$ (Fig. 4a), and then we employed PGD to remove self-connections, enforce Dale’s principle with a 4:1 Ex:In ratio, and set a sparsity $sp = 40\%$ (defined as the percentage of weights equal to zero). The PGD steadily reduced the loss ${\mathcal{L}}$, reaching a negligible value, provided that the network had enough neurons (Fig. 4b). It is remarkable that so dissimilar synaptic weight matrices, like the ones depicted in Fig. 4a,c,d, gave rise to the same stimulus–response mapping.

We noted that structural constraints could not be imposed on networks with low number of neurons, for example, networks with $N = N_{in} + N_{rec} < 2M$. This is not surprising, since it is expected that imposing more constraints require more parameters. To evaluate the efficiency of the PGD in relation with the number of neurons, we imposed the above structural constraints for networks that solved the s-task with $\tau = 3$ to $\tau = 6$, and $N_{rec}$ between 32 and 256 neurons. Since matrix **U** and vector ${{\varvec{\uptheta}}}$ were randomly chosen, it was expected that some of them resulted in matrices **W** for which the structural constraints were impossible to apply. Consequently, we measured the efficiency of PGD by computing *# attempts*, the number of networks that were required until obtaining the first successfully constrained network. It can be seen that *# attempts* decreased as the number of neurons increased (Fig. 4e). Concordantly, the computing time required to obtain a successfully constrained network decreased as the number of neurons increased (Fig. 4f), probably due to the decrement in *# attempts* that were required. The fitting time was higher for networks with the highest neuron count, but always within the order of tens of seconds, even for $N_{rec} = 256$.

### Exploiting the FTP algorithm for hypothesis testing

A distinctive aspect of the FTP algorithm is that the desired dynamic of the network is defined first and in great detail. In fact, we can specify the network dynamic up to the level of single state transitions, and the algorithm finds the set of synaptic weights that makes the desired dynamic possible. This approach is ideal for testing very specific hypotheses about the relationship between network function (neural dynamics and task performance) and the underlying structure (synaptic connectivity).

**Figure 5:**
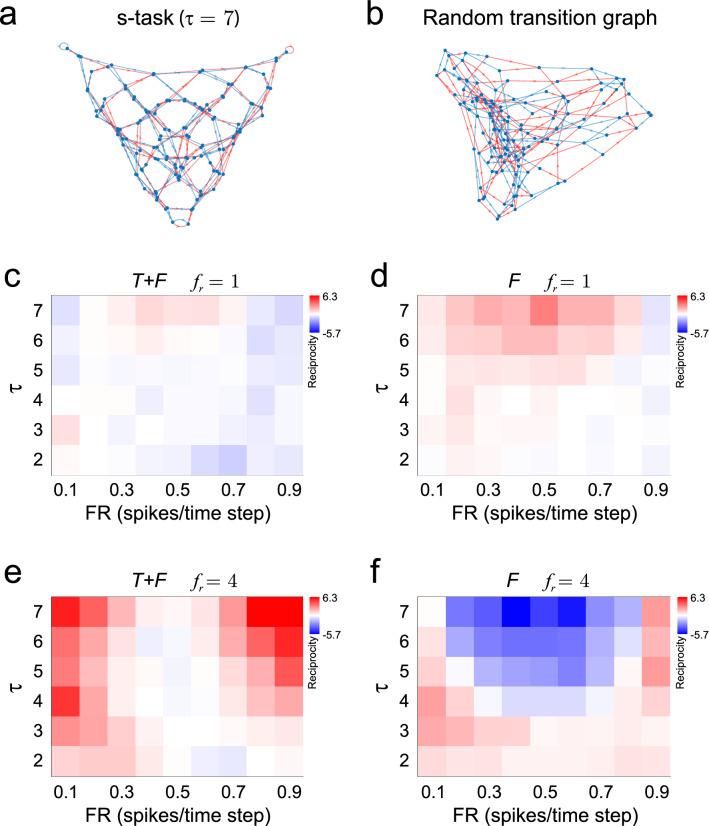
Reciprocity as a function of $\tau$, FR and the type of transition graph. (**a**) Transition graph for solving the s-task with $\tau = 7$. Blue and red lines represent transitions gated by $s_{1}$ and $s_{2}$, respectively. (**b**) Random transition graph. Nodes (network states) may receive different number of incoming connections. There are 24 nodes that are gated by both stimuli. (**c**) Reciprocity for *T* + *F* networks, with $f_{r} = 1$, as a function of $\tau$ and target FR. Reciprocity changes from slightly negative to slightly positive as $\tau$ increases. For $\tau = 7$, reciprocity is maximized around target FR = 0.5 spikes/time step and decreases for lower and higher values of target FR. (**d**) *F* networks with $f_{r} = 1$ shows increasing positive reciprocity as $\tau$ increases, maximized at target FR = 0.5 spikes/time step. (**e**) When the number of neurons is higher ($f_{r} = 4$), *T* + *F* networks show positive reciprocity that is minimal around target FR = 0.5, and increases towards higher and lower target FR, reaching the highest reciprocity values among all networks screened. (**f**) Reciprocity of *F* networks turns increasingly negative as $\tau$ increases, reaching the lowest reciprocity among all networks screened, around target FR = 0.5 spikes/time step. For all panels, 30 networks were constructed for each $\tau$ and target FR combination. Normalized means (mean/SD) are shown. Positive and negative reciprocity values were mapped separately to colours red and blue, respectively. Red tones go from 0 reciprocity (white) to maximal positive reciprocity (pure red). Blue tones go from 0 reciprocity (white) to maximal (in absolute value) negative reciprocity (pure blue). All random graphs were constructed with $f_{bc} = 0.5$. Graphs were plotted with the Force-directed layout.

We wanted to understand how the firing rate and firing dynamic constrained connectivity. To that end, we constructed networks that solved the s-task, over a range of network sizes and mean firing rate, and compared them with networks that had the same number of neurons and network states but for which the graphs of transitions between network states were generated at random (Fig. 5a,b). These “random transition” networks did not have sequence memory, but their dynamic was similar in complexity to that of networks that solved the s-task, turning them in perfect examples to study the connectivity features behind the capacity to codify sequences of stimuli. We focused on measuring the level of reciprocity in the network, which has been observed experimentally^21^ and its implications studied theoretically^22^. By screening networks with memory ranging from $\tau = 2$ to $\tau = 7$, and FR from 0.1 spikes/time step to 0.9 spikes/time step, we found that reciprocity varied with $\tau$, FR and neuron number. In particular, we observed that, when $f_{r} = 1$, reciprocity was positive and of lower mean for networks that were the minimum Frobenius norm solution to the s-task (*T* + *F* networks, Fig. 5c), in comparison with networks that were the minimum Frobenius norm solution to a random transition graph (*F* networks, Fig. 5d). However, for bigger *f*_*r*_ the relationship was inverted, and *T* + *F* networks showed positive reciprocity (Fig. 5e) while *F* networks showed negative reciprocity (Fig. 5f).

**Figure 6:**
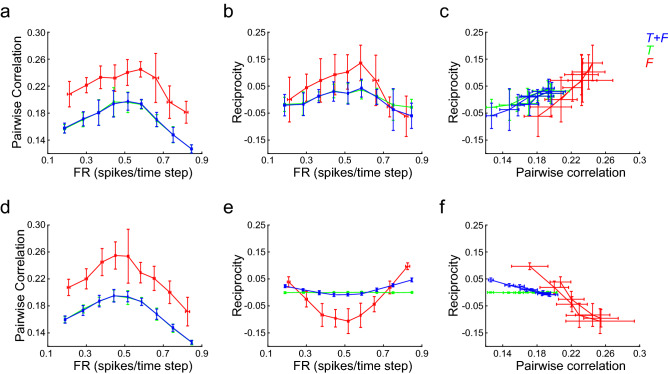
Correlation and reciprocity differentiate networks with sequence memory from random transition networks. (**a–c**) Networks constructed to solve the s-task with $\tau = 7$ and $f_{r} = 1$ (*T* + *F* networks), their isofunction network (*T* network), and networks with the same number of neurons and network states that follow a random transition graph (*F* networks). (**a**) Correlation increases as FR approaches 0.5 spikes/time step. *F* networks show a positive offset in comparison with *T* + *F* and *T* networks. (**b**) The dependency between reciprocity and FR is similar to the dependency between CC and FR. Higher reciprocity values are found in *F* networks. (**c**) Reciprocity grows linearly with correlation, as expected from panels (**a**) and (**b**). (**d**–**f**) idem **a–c**, but with $f_{r} = 4$. (**d**) The CC/FR relationship is similar to the one observed with a lower neuron number (panel (a)). (**e**) The reciprocity/FR relationship inverted as the neuron number was increased. Reciprocity is minimized as FR approaches 0.5 spikes/time steps. *F* networks show pronounced negative reciprocity. (**f**) Reciprocity decreases linearly with correlation, as expected from panels (**d**) and (**e**). Mean ± SD are shown; n = 10 networks were constructed for each target FR and network type. All random graphs were constructed with $f_{bc} = 0.25$.

To further describe these relationships, we selected networks constructed for $\tau = 7$ and $f_{r} = 1$ (Fig. 6a–c) and $f_{r} = 4$ (Fig. 6d–f). Signal correlation varied with FR following an inverted U-shape relationship, with a maximum next to 0.5 spikes/time step (Fig. 6a,d). Note that, up to FR = 0.5 spikes/time step, CC increased with FR, as has been observed experimentally^23,24^. Interestingly, the way CC changed with FR was similar for both *T* + *F* and *F* networks, with the distinction that *F* networks had overall higher correlations than *T* + *F* networks. Shuffling the inter-spike interval of each neuron destroyed the dependence between FR and CC. The CCs after shuffling were also much smaller than the CC values of the non-shuffled firings (CC_shuffled_ = 0.0224 ± 1.10^–4^, mean ± SD, for n = 90 T + *S* networks pooled over all FR values). These results ruled out the possibility that correlations were trivially increasing with FR because of the higher number of spikes. We also found an inverted U-shape relationship between reciprocity and FR, and a linear relationship between reciprocity and CC. With $f_{r} = 1$ reciprocity tended to be maximized as FR approached 0.5 spikes/time step, with *F* networks showing higher (and positive) reciprocity (Fig. 6b). With $f_{r} = 4$, reciprocity tended to increase as FR departed from 0.5 spikes/time step towards lower and higher values, i.e., networks with lower correlation (Fig. 6e). Specially, *F* networks showed negative reciprocity for all firing rates, except for the more extreme cases (0.1 and 0.9 spikes/time step). Just like the reciprocity/FR relationship inverted with the number of neurons, so did the reciprocity/CC relationship. Networks with higher reciprocity had higher correlation when the number of neurons was low (Fig. 6c). However, and somewhat counterintuitive, when the number of neurons was higher, more reciprocity implied lower correlation (Fig. 6f). Networks that solved the s-task but did not minimize the Frobenius norm (*T* networks) showed almost zero reciprocity, implying that reciprocity was not a property of all networks that solve the s-task. On the contrary, most networks that solved the s-task did not show significant reciprocity, unless other structural constraints, such as Frobenius norm minimization, was imposed. However, Frobenius norm minimization alone only produced negative reciprocity (in random graphs). For positive reciprocity to occur in networks with high number of neurons, both high sequence memory and Frobenius norm minimization were required.

**Figure 7:**
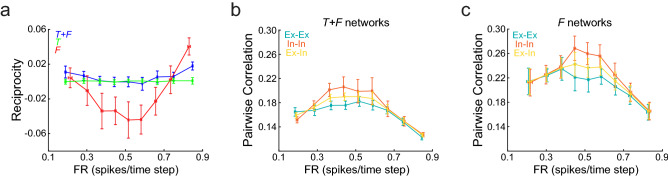
Reciprocity and correlation of structurally constrained networks. (**a**) Reciprocity as a function of FR for networks without self-connections and Dale’s principle with 1:1 Ex:In ratio. Reciprocity shows a parabolic relationship with FR, decreasing as FR approaches 0.5 spikes/time step. *F* networks show strong negative reciprocity, while *T* networks reciprocity is close to zero. (**b**) Pairwise correlation for *T* + *F* networks as a function of firing rate. The correlation was computed over pairs of excitatory neurons (Ex-Ex), pairs of inhibitory neurons (In-In), and pairs of one excitatory and one inhibitory neuron (Ex-In). Correlation has a maximum close to 0.5 spikes/time step. The In-In pairs show the highest correlations, followed by the Ex-In pairs. The Ex-Ex pairs show the lowest correlation. (**c**) pairwise correlation for *F* networks as a function of firing rate. The CC/FR relationship is similar to the one observed for *T* + *F* networks, although *F* networks correlation is displaced towards higher values. Mean ± SD are shown; n = 20 networks were constructed for each target FR and network type. Firing rates of excitatory or inhibitory neurons are displayed for Ex-Ex and In-In curves, respectively. For Ex-In curves, the average FR over all neurons is shown.

We asked whether the results depicted in Fig. 6 could be replicated in networks that lacked self-connections and complied with Dale’s principle. To that end we imposed these structural constraints to networks constructed with $\tau = 7$ and $f_{r} = 4$, and found a reciprocity/FR relationship that resembled the one observed in unconstrained networks, with *F* networks showing prominent negative reciprocity and *T* + *F* networks showing increasing reciprocity as FR departed from 0.5 spikes/time step (Fig. 7a). Correlations increased as FR approached 0.5 spikes/time step, with *F* networks showing more correlation than *T* + *F* networks (Fig. 7b,c). Correlation in *T* + *F* and *F* networks were higher for pairs of inhibitory neurons than for pairs of excitatory neurons, as has been observed experimentally^25^, while correlations between excitatory and inhibitory neurons lied in the middle.

**Figure 8:**
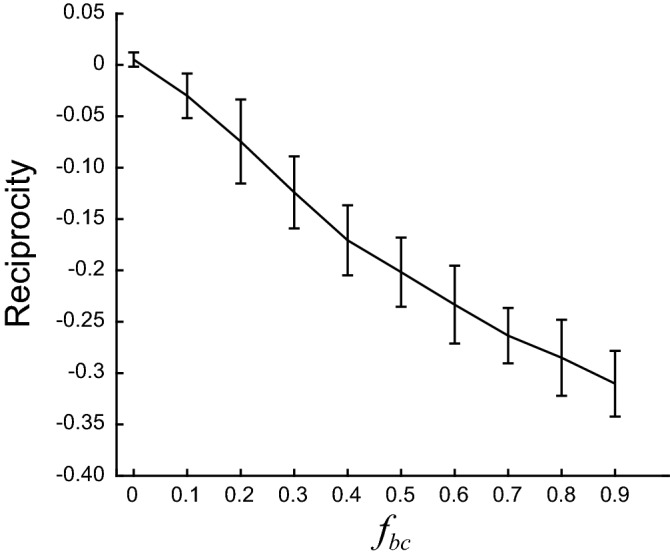
Relative-coding network states cause negative reciprocity. Reciprocity as a function of $f_{bc}$, the fraction of bicolored nodes, in networks that follow random transition graphs. Reciprocity decreases linearly with $f_{bc}$, approaching zero as $f_{bc}$ approaches zero. Mean ± SD are shown, n = 30 networks for each $f_{bc}$. Networks were constructed with target FR = 0.5 spikes/ time step, and with the same number of neurons and network firing states as *T* + *F* networks constructed with $\tau = 7$ and $f_{r} = 4$.

We went further on by asking which features of network dynamics were responsible for the differences in reciprocity that we observed between *T* + *F* and *F* networks. The FTP algorithm finds the network connectivity from a detailed description of the network dynamics, specified in its transition graph. Therefore, we hypothesized that specific changes in the transition graph could have a precise impact in network reciprocity. In *T* + *F* networks, any given network state could only be reached after the presentation of either *s*_1_ or *s*_2_, but not both. This is clearly visualized in the transition graph of Fig. 1b, in which each node has all blue or all red incoming edges, but never incoming edges of both colours. These network states codify stimuli in an absolute manner since it only suffices to know the network state at time step *t* to know the identity of the presented stimuli at that time step. We termed these nodes *monocoloured* nodes. On the other hand, in an *F* network each network state can be reached from either *s*_1_ or *s*_2_, or from both. Network states which can be reached from both stimuli (termed *bicoloured* nodes) codify stimuli in a relative manner, meaning that the identity of the stimulus presented at time step *t* can be decoded only if *both* the network states at *t* and $t - 1$ are known. Then, we specifically asked whether the proportion of bicolored and monocoloured nodes could explain the strong differences in correlation and reciprocity found between *T* + *F* and *F* networks. To that end, we constructed *F* networks with increasing fraction of bicolored nodes (*f*_*bc*_), and computed reciprocity for fixed $\tau$, FR and *f*_*r*_ (Fig. 8). We found that reciprocity became increasingly more negative as we increased the fraction of bicolored nodes. When all network states were monocoloured ($f_{bc} = 0$), networks showed zero reciprocity, as observed in *T* + *F* networks with the same FR and *f*_*r*_. This result highlights the power of the FTP algorithm to relate neural dynamics with network connectivity.

## Discussion

We have presented a simple method to generate binary neural network models that accomplish the desired task. Networks composed of binary neurons are computationally inexpensive, and despite their simplicity many neurophysiological and neuroanatomical observations have been recapitulated employing these networks^22,26^. Our key contribution is to note that, for networks in which neuron inputs are linearly added, their synaptic weights can be found by solving a system of linear equations. In turn, this system can be constructed from the transition graph associated with the solution of the target task. System consistency is guaranteed if the dependent variables of the system (the neuron activations) are linearly combined following the linear dependences among the independent variables (the firing states). We have shown how the FTP algorithm works with the simplest of networks. Yet, we think that the same procedure could be implemented in networks composed of more complex neuron models, like the firing rate model or the leaky integrate-and-fire model, provided that a system of linear equations can be constructed.

Current automated methods of network model construction rely on off-the-shelf optimization algorithms typically employed in the artificial intelligence field, like stochastic gradient descent^5^, genetic algorithms^27^, or evolutionary strategies^28^. These optimization algorithms iteratively change network parameters in a direction that minimizes a loss function and have proved to be very effective in finding networks that solve very complex tasks^29,30^. However, they require a considerable amount of human intervention, and there is no certainty that they will arrive at a solution. Moreover, each optimization iteration requires the evaluation of the network, which is time consuming, especially for a recurrent network performing in a multi-trial task. In contrast, the FTP algorithm reduces the problem of finding a suitable network to a series of linear combinations and the solution of a linear system, with both operations performed in polynomial time. Most importantly, it is guaranteed that the resulting network will solve the task perfectly.

Traditional optimization algorithms require the definition of a loss function that encompasses all the constraints the network should satisfy, whether these are task related, activity related, or structural. Then, the loss function is minimized and hence all constraints are enforced at once. In this scenario, the relationship between parameters and the loss function can be quite complex, and conflict between constraints may emerge. Conversely, one key advantage of our method is that it uncouples the dynamic and coding aspects of the network from the structural aspects, giving us the opportunity to adjust them independently. Moreover, since the method proceeds from the network firing states to its parameters, it allows us to find networks with desired activity profiles, and to study the resulting connectivity. Further structural constraints can be enforced in a second stage, by projected gradient descent, or any other optimization algorithm. The fact that projected gradient descent worked so well suggests that structural constraints are easy to implement once the connectivity required to solve the task is in place. This is probably because many connectivity features of a network, such as its sparseness or its clustering coefficient, are a simple function of its synaptic weights. On the contrary, the relationship between the synaptic weights and the network function, or coding capabilities, is far more complex. In this way, our method gives more control over each class of constraint, making the whole process simpler at the same time.

To show its applicability, we employed the FTP algorithm to construct networks that solve a stimuli sequence memory task (s-task) in which networks have to codify in their network firing states the sequence of the last $\tau$ stimuli that were presented. The s-task, akin to the n-back task commonly employed in cognitive neuroscience, is relevant within the scope of working memory function. Working memory is traditionally associated with maintaining information about a single stimulus in the persistent activity of recurrently connected neurons^31,32^, although mounting evidence suggests that neuron populations code information in the form of highly heterogenous firing sequences^33,34^. Sustained activity can be a suitable strategy when there is one specific relevant stimulus to attend, whose identity has been already elucidated. However, more complex scenarios require keeping track of sequences of stimuli. An example of this case is the processing of language, in which the succession of utterances must be integrated over time, from phonemes to words, to phrases, so that the meaning of speech depends on the whole sequence^35^. We explored the case of two stimuli presented with equal probability, but the analysis could be extended to more realistic cases in which the stimulus distribution is not uniform. It is expected that statistical regularities in the sequences of stimuli are going to be exploited by the network, resulting in more specialized connectivity. The relationship between sequence statistics and network structure should be further studied. For example, it would be of interest the case in which stimuli last more than one time-step, and they are interleaved by another set of stimuli that act as distractors. Then, the relationship between feedback and feedforward connections could be studied, in relation with the duration of each stimulus presentation, and with that of the distractors.

The structure–function relationship is central to neuroscience^36–38^. Connectivity at the macro, meso, and micro scale, neuron biophysics, plasticity mechanisms, among other structural traits, all act co-ordinately to give sophisticated adaptive behaviour. It is widely believed that the structural properties of networks have evolved to proficiently perform a function, many times in an optimal way^39,40^. However, brain structure could also be the result of other constraints, different from those imposed by adaptive behaviour. For example, neural network modularity might have emerged as an adaptative structural trait for solving tasks that have a modular or hierarchical component^41^, but it could also have emerged as the result of previously acquired structural traits, such as constraints in the length of dendrites and axons, which preclude the possibility of a much wider connectivity. Thus, determining how much of the structure observed in the brain comes from task-related constraints and how much comes from other structural traits is central to understanding the structure/function relationship. A theoretical approximation to this issue consists on constructing neural network models that solve different kinds of tasks under a variety of structural constraints, and then study the patterns of connectivity that emerge and relate them to the observed connectivity in the brain. This approximation requires to sample as uniformly as possible from the set of networks that fit both the task and the structural constraints. However, optimization methods commonly employed in network parameter fitting may give a restricted set of solutions, thus biasing any conclusion about the structure/function relationship. Another issue is that some connectivity traits could emerge only in networks of certain size, and fitted to several tasks. In this case, fitting large networks with complex cost functions could have a high computational cost. Consequently, generating a relatively large sample of networks suitable for statistical treatment of their connectivity could result unfeasible. On the other hand, the FTP algorithm is very well suited for answering structure/function questions, since exact solutions can be computed starting from an arbitrary set of population firing states, as long as they define a system of equations that has a solution.

Hypotheses that link structure and function can be tested with the help of the FTP algorithm, by constructing networks that follow transition graphs that instantiate some null hypothesis. Following this approach, we constructed networks that had the same number of network states and neurons required to solve the s-task, but whose state transitions were chosen at random. With this tool at hand, we were able to show how network reciprocity depended on the memory demand and the size of the network. The same procedure can be followed to build any other set of networks under some relevant null hypothesis. Such networks can be easily constructed with the FTP algorithm, while they would be hard to construct with regular optimization algorithms.

Evidence for high reciprocity has been found experimentally, by measuring excitatory postsynaptic potentials of reciprocally connected neurons *in vitro*^21^. It has also been the centre of theoretical analysis. For example, it has been shown that high reciprocity is recapitulated in networks of binary neurons that have a maximum number of attractors^22^. Interestingly, the same work showed that reciprocity is lost when networks are optimized to store sequences of uncorrelated network states. However, we found that networks of high reciprocity are capable of displaying long firing state sequences when their dynamics codify the sequences of previously presented stimuli. Therefore, our work complements previous studies which have shown that reciprocity is one of the key connectivity features that support the computation performed by the neocortex.

Reciprocity was absent in networks taken at random from the set of all connectivities that solve the s-task (the *T* networks in Fig. 6). This implies that the observed reciprocity is the result of solving the s-task with the additional constraint of weights minimizing the Frobenius norm, the latter being understood as the consequence of an upper bound on the number of receptors and vesicles in a synapse. Thus, to explain one structural feature (reciprocity), a functional feature (solving the s-task), and another structural feature (Frobenius norm minimization) were required. It would be interesting to study to what extent other structural features encountered in biological neural networks, like modularity or sparsity of connections, can be explained as the answer to some computational demand of adaptive behaviour, or as the byproduct of another structural feature, or as the interaction of both factors, as is the case with the s-task.

In conclusion, we have provided an algorithm that inverts the usual process by which neural networks are constructed. It can be employed to probe the dependency between the firing statistics, connectivity, and function of a network in a way that is not matched by current optimization algorithms. Moreover, it is computationally inexpensive. For these reasons, we consider the FTP algorithm to be a powerful alternative method for constructing neural network models of brain function.

## Methods

### Nomenclature

Vectors are represented with bold lowercase letters and are considered row vectors. Matrices are represented with bold uppercase letters. In Table 1 we have summarized the principal symbols employed throughout the paper, together with their description.

**Table 1:** Symbols with descriptions.

Symbol	Description
**y**	Binary row vector of length *N*_*in*_. Represents the firing state of input neurons. Codifies a stimulus
**z**	Binary row vector of length *N*_*rec*_. Represents the firing of the recurrent network
**u**	Real-valued row vector of length *N*_*rec*_. Represents the activation states of neurons in the recurrent network
**W**^*in*^	Matrix of synaptic weights from sensory neurons to the recurrent network. Columns are incoming connections
**W**^*rec*^	Matrix of synaptic weights among neurons of the recurrent network. Columns are incoming connections
*N*_*in*_	Number of input neurons ($N_{in} = 2$ throughout this work)
*N*_*rec*_	Number of neurons in the recurrent network
***N***	Total number of neurons (input and integration)
***M***	Total number of network firing states
${{\varvec{\uptheta}}}$	Row vector of neuron’s thresholds
**c**	Row vector, obtained after concatenating one **y** vector with one **z** vector
**C**	Matrix whose rows are **c** vectors. Coefficient matrix in a system of linear equations
**U**	Matrix composed of row vectors **u**. Contains activation states reached by the network from the firing states in matric **C**
**W**	Matrix resulting from concatenating matrices **W**^*in*^ and **W**^*rec*^
**U**_*base*_	Matrix of row vectors **u** picked at random
**U**_*lc*_	Matrix composed of the rows in **U**_*base*_ and linear combinations thereof. There is one row for each network state
**Z**	Binary matrix, obtained by applying threshold ${{\varvec{\uptheta}}}$ to matrix **U**_*lc*_
${{\varvec{\Delta}}}$	Real-valued vector of length *N*_*rec*_. Each component is the difference between activations after *s*_1_ and *s*_2_ presentation, when starting from the same network firing state
***f***_***r***_	Redundancy factor: quotient between the number of neurons and the number of sequences codified in an s-task
***f***_*cc*_	Multiplying factor to induce signal correlation
***f***_*bc*_	Number of network states reachable after *s*_1_ or *s*_2_ presentation, divided by the total number of network states

### Attaining system consistency in the s-task

We consider a network that solves an s-task, with 2 stimuli and sequences of length $\tau$. Therefore, the network has to display at least a number of network states $M = 2^{\tau }$. If we number the network states and sort transitions as in Fig. 1b–d, we have that, if Eq. (3) has a solution, rows in $[{\mathbf{C}}\,\,\,{\mathbf{U}}]$ (the augmented matrix) should satisfy:

6 $$[{\mathbf{y}}_{2} \,\,\,{\mathbf{z}}^{T + 1} \,\,\,{\mathbf{u}}^{T + 1} ] = [{\mathbf{y}}_{1} \,\,\,{\mathbf{z}}^{T} \,\,\,{\mathbf{u}}^{T} ] - [{\mathbf{y}}_{1} \,\,\,{\mathbf{z}}^{P} \,\,\,{\mathbf{u}}^{P} ] + [{\mathbf{y}}_{2} \,\,\,{\mathbf{z}}^{P + 1} \,\,\,{\mathbf{u}}^{P + 1} ]$$

where *T* values are odd numbers between 1 and $2M - 1$ such that $[{\mathbf{y}}_{1} ,{\mathbf{z}}^{T} ]$ and $[{\mathbf{y}}_{2} ,{\mathbf{z}}^{T + 1} ]$ are the *T* and *T* + 1 rows of **C**, and ${\mathbf{u}}^{T}$, ${\mathbf{u}}^{T + 1}$ are the *T* and $T + 1$ rows of **U**. The row index *P* is and odd number between 1 and $2M - 1$. Note that ${\mathbf{z}}^{T} = {\mathbf{z}}^{T + 1}$ and ${\mathbf{z}}^{P} = {\mathbf{z}}^{P + 1}$, but ${\mathbf{u}}^{T} \ne {\mathbf{u}}^{T + 1}$ and ${\mathbf{u}}^{P} \ne {\mathbf{u}}^{P + 1}$. Equation (6) shows us how row vectors in matrix **U** should be linearly combined such that Eq. (3) has a solution. We have that:

7 $${\mathbf{u}}^{T + 1} = {\mathbf{u}}^{T} - {\mathbf{u}}^{P} + {\mathbf{u}}^{P + 1}$$

This means that the number of linear combinations in **U** is $R = 2^{\tau } /2 - 1$, and ${\text{rank}} ({\mathbf{U}}) = 2^{\tau } /2 + 1$. Note that rewriting Eq. (7) we have:

8 $$u_{{m,s_{2} ,i}} - u_{{m,s_{1} ,i}} = \Delta_{i}$$

where $u_{{m,s_{1} ,i}}$ and $u_{{m,s_{2} ,i}}$ are the activations that neuron *i* adopts after the presentation of *s*_1_ and *s*_2_, and coming from a network state *m*. In other words, Eq. (8) tells that the difference in effects provoked by the stimuli is a constant for each neuron, regardless of which network state or transition we are dealing with. This fact is not surprising, since synaptic weights are held fixed, so each stimulus has the same effect at any time, which is specific for each neuron. Thus, making the system of equations in (3) consistent is equivalent to guarantee that activation values are chosen so that each stimulus has a constant effect on the synaptic activations.

To construct matrix **U** we first defined a vector of thresholds $${{\varvec{\uptheta}}}$$ with elements $$\theta_{i} \in \left\{ {{\raise0.5ex\hbox{$\scriptstyle 1$} \kern-0.1em/\kern-0.15em \lower0.25ex\hbox{$\scriptstyle 2$}},{\raise0.5ex\hbox{$\scriptstyle 3$} \kern-0.1em/\kern-0.15em \lower0.25ex\hbox{$\scriptstyle 2$}},{\raise0.5ex\hbox{$\scriptstyle 5$} \kern-0.1em/\kern-0.15em \lower0.25ex\hbox{$\scriptstyle 2$}}} \right\}$$. Then, we constructed base matrix **U**_*base*_ with $$M_{base} = 2^{\tau } /2 + 1$$ row vectors such that:

$${\mathbf{U}}_{base} (m,i) = \theta_{i} + r(m,i) + \frac{1}{2}$$

where $r(m,i)$ is an integer uniformly sampled from the [− 5,5] interval. We added the term 1/2 to avoid fitting errors when numerically solving the system, otherwise the activation values could be equal to the threshold values, which would result in erroneous firing states because of numeric precision issues. We chose a uniform distribution over integers for simplicity, although any other distribution could be employed. The same goes for the threshold values.

This initial randomly generated matrix **U**_*base*_ is required to be full rank. We computed the vector ${{\varvec{\Delta}}}$ of $\Delta_{i}$ elements as the difference between the first two rows of **U**_*base*_. Next, we applied Eq. (8) to generate the remaining *R* rows as linear combinations of the third to the last row of **U**_*base*_, obtaining $2^{\tau }$ row vectors that constitute the matrix **U**_*lc*_. Each row vector **u** in matrix **U**_*lc*_ had the neuron activations for one of the $2^{\tau }$ network states. Applying Eq. (8) creates a dependency between $u_{{m,s_{1} ,i}}$ and $u_{{m,s_{2} ,i}}$. Hence, for each linear combination we chose at random which activation value (the one associated with *s*_1_ or *s*_2_) was defined in terms of the other. This was to ensure that *u* value distributions were equal for both stimuli. We constructed matrix **Z** by applying threshold ${{\varvec{\uptheta}}}$ to **U**_*lc*_, and then we followed the ordering depicted in Fig. 1b–d to construct matrix **U** from **U**_*lc*_, and matrix **C** from **Z** and vectors **y**_1_, **y**_2_. Finally, we employed Eq. (4) to obtain the synaptic weight matrix **W**. Since matrix **W** is the minimum Frobenius norm solution to Eq. (4) and defines a network that solves the s-task, we say that **W** defines a *Task* + *Frobenius* (*T* + *F)* network.

The algorithm described above works under the assumption that two conditions are met after thresholding: 1) the resulting vectors **z** are all different, and 2) they are linearly independent. If thresholding **U**_*lc*_ gives vectors **z** that appear more than once, this would result in lower performance in the task, since not all sequences of length $\tau$ will be encoded. On the other hand, if linear independency fails after thresholding, then matrix **C** will have more linear combinations than the contemplated in Eq. (5), meaning that combining the rows of **U** following Eq. (8) will not be enough, and some linear dependencies in **C** will be lost in the augmented matrix, making the system inconsistent. In our implementation of the algorithm, if any of these two conditions were not verified, then the algorithm was restarted from the beginning. This occurred with low probability, for $\tau < 5$. For higher $\tau$, both conditions were always fulfilled in one attempt.

In the previous explanation we assumed that $N_{rec} = 2^{\tau }$, such that there is one neuron per sequence of length $\tau$. It was possible to fit networks with lower number of neurons, but undesired linear dependencies in **C** after thresholding, or a number of network states bellow $2^{\tau }$ occurred with higher probability, especially for $\tau > 3$.

### Network simulation and synaptic weights statistics

Networks were evaluated in the s-task during at least $N_{iter} = 10.2^{\tau }$ time steps, to gather enough samples of each network state. To assess the similarity between the synaptic weight distribution and a normal distribution we computed the Kolmogorov–Smirnov two samples statistic, between the set of synaptic weights and a set of normally distributed values of the same mean, variance, and sample size than that of the synaptic weights.

Equation (4) gives the matrix **W** with lowest Frobenius norm^12^. Since we are considering networks with $N = 2^{\tau } + 2$ total neurons (including input and integration neurons), there are infinite solution weight matrices for the same system of equations defined by **C** and **U**. These solutions lay in a subspace of ${\mathbb{R}}^{N}$, of dimension $N - {\text{rank}} ({\mathbf{C}})$. The set of all solutions can be obtained by computing the sum between **W** and a matrix ${\mathbf{\Delta W}}$ that satisfies:

9 $${\mathbf{C}}\,{\mathbf{\Delta W}} = {\mathbf{0}}$$

10 $${\mathbf{\Delta W}} = \ker ({\mathbf{C}}){\mathbf{\mathcal{M}}}$$

where $\ker ({\mathbf{C}})$ is an orthonormal basis of the null space of **C**, of dimensions $N{\mkern 1mu} {\text{x}} {\mkern 1mu} (N - {\text{rank}} ({\mathbf{C}}))$, **0** is a matrix of zeros, and ${\mathbf{\mathcal{M}}}$ is a linear mapping of dimensions $(N - {\text{rank}} ({\mathbf{C}})){\text{x}}{\mkern 1mu} N_{rec}$. This set of solutions share the same stimulus–response mapping. We say they conform an isofunction space.

In several occasions, networks with $N_{rec} > 2^{\tau }$ were desired. Hence, we defined $N_{rec} = 2^{\tau } f_{r}$, where $f_{r}$ stands for ‘redundancy factor’, as the network has *f*_*r*_-times more neurons than required to solve the s-task with that specific $\tau$.

### Computation of fitting times for FTP and SGD

We assessed the efficiency of the FTP algorithm and an SGD algorithm by measuring the time required to find networks of $N_{rec} = 1024$ neurons that solve an s-task with $\tau = 1$ to $\tau = 10$. Since in our neuron model the firing state is a non-differentiable function of the activation states, we relied on a surrogate derivative:

$$\frac{\partial z}{{\partial u}}: = \max \{ 0,1 - \left| \upsilon \right|\}$$

where $\upsilon = u - \theta$. We took this surrogate derivative from Bellec et al.^15^, although we obtained better results not dividing by the threshold, as originally proposed. One response neuron was added, whose output at a given time step $t$ was defined as $o_{t} = \tanh ({\mathbf{w}}^{{{\mathbf{out}}}} {\mathbf{z}}_{t} )$, where ${\mathbf{w}}^{{{\mathbf{out}}}}$ contains the synaptic weights between integration and output neurons. The loss function to minimize was the quadratic error $E = (o_{target} (s) - o)^{2} /2$, with $o_{target} (s_{1} ) = 1$ and $o_{target} (s_{2} ) = - 1$. We implemented back propagation thought time (BPTT), unfolding the network in sequences of 30 timesteps and taking minibatches of 30 sequences. We achieved best performance by initializing **W**^*in*^ and **w**^**out**^ with zeros, while matrix **W**^*rec*^ was initialized with samples from a standard normal distribution and then dividing it by its eigenvalue of higher absolute value. Adaptive learning rates were implemented thought *Adam*^14^, with parameters $\beta_{1} = 0.9$, $\beta_{2} = 0.999$, $\varepsilon = 10^{ - 8}$ and $\alpha = 10^{ - 4}$. Normalizing gradients to unit length also proved to be helpful in accelerating convergence. Networks were trained until the mean error over the last 10 minibatches was below 0.01. After training, networks were tested with 10,000 stimuli presentations. The maximum testing error across all $\tau$ values was 0.03.

### Imposing activity constraints

To construct networks with desired FR we generated **U**_*base*_ as previously described, but adjusted the sign of $r(m,i)$ such that, after thresholding, matrix **C** had a fraction of ones that equalled the target FR. To induce signal correlation, we scaled vector ${{\varvec{\Delta}}}$ by a factor *f*_*cc*_. This manipulation causes neurons to have very different firing rates for *s*_1_ and *s*_2_, leading to an increased signal correlation. By following this procedure, we constructed networks in Fig. 3. The target FR values were taken from the range between 0.1 to 0.9 spikes/time step, in steps of 0.1 spikes/time step. The *f*_*cc*_ values were taken from the range between 1 and 10 in unitary steps. A total of 30 networks were constructed for each combination of FR and *f*_*cc*_ values within those ranges. For each network, the average FR was computed over the FR of all neurons in the network. Similarly, the average correlation coefficient (CC) was computed from the Spearman correlation coefficient computed for all neuron pairs.

We also employed a genetic algorithm (GA) to evolve a population of matrices **U**_*base*_ to adjust their mean FR and absolute CC. We employed a population of $N_{pob} = 200$ individuals, each one composed of one matrix **U**_*base*_ and one vector ${{\varvec{\uptheta}}}$. For each individual we constructed matrices **U** and **C**, and computed an approximate value of FR and correlation, under the assumption that each network firing state occurred with equal probability. The fitness *F* of an individual was computed as:

$$F = 1 - \frac{{\left| {FR - FR_{target} } \right| + \left| {\left| {CC} \right| - \left| {CC_{target} } \right|} \right|}}{2}$$

where *FR* and *CC* are the firing rate and correlation computed over the network firing states, and $FR_{target}$ and $CC_{target}$ are the firing rate and correlation we want the network to have. If an individual produced an inconsistent system, or a system with not enough network states, its fitness was set to zero. We picked the $T = 0.1N_{pob}$ individuals with the highest fitness as parents. Then, we picked parents at random and mutated ${\mathbf{U}}_{base}$ by adding gaussian noise to each matrix element, of zero mean and standard deviation $\sigma = 0.1$. One of the individuals of each generation was an unmutated copy of the best individual of the previous generation (elitism). Threshold vectors ${{\varvec{\uptheta}}}$ were not mutated. The GA was run until the average fitness surpassed $F_{target} = 0.95$. Firing rates and correlations shown as black dots in Fig. 3d were computed by running the network constructed from the elite ${\mathbf{U}}_{base}$ during $30.2^{\tau }$ time steps.

### Imposing structural constraints

Solving Eq. (4) gives networks with minimum Frobenius norm. These networks do not comply with basic structural features observed experimentally, such as the low probability of self-connections, or Dale’s principle. To impose such structural features, we constructed a matrix ${\mathbf{\Delta W}}_{d}$ such that ${\mathbf{W}}_{d} = {\mathbf{W}} + {\mathbf{\Delta W}}_{d}$. Matrix **W**_*d*_ is a matrix which fulfils the desired structural constraints. Most probably ${\mathbf{\Delta W}}_{d}$ is not within the null space of **C**, and thus **W**_*d*_ will not be a solution to the system defined by **C** and **U**. Hence, we defined a matrix:

11 $${\mathbf{\Delta W}} = \ker ({\mathbf{C}}){\mathbf{\mathcal{M}}}_{sc}$$

where ${\mathbf{\mathcal{M}}}_{sc} = \ker ({\mathbf{C}})^{ + } {\mathbf{\Delta W}}_{d}$, and $\ker ({\mathbf{C}})^{ + }$ is the Moore–Penrose pseudoinverse of $\ker ({\mathbf{C}})$. Matrix ${\mathbf{\mathcal{M}}}_{sc}$ is a linear mapping that incorporates the desired structural constraints, making ${\mathbf{\Delta W}}$ the change in matrix **W** within the null space of **C** that is closest to ${\mathbf{\Delta W}}_{d}$, in the least squares sense.

We imposed three structural constraints: no self-connections, Dale’s principle, and a certain degree of sparsity. Thus:

$${\mathbf{\Delta W}}_{d} = {\mathbf{\Delta W}}_{self} + {\mathbf{\Delta W}}_{Dale} + {\mathbf{\Delta W}}_{sp}$$

The $(i,j)$ element in matrix ${\mathbf{\Delta W}}_{self}$ (which deletes self-connections in the integration neurons) was defined as:

$${\mathbf{\Delta W}}_{self} (i,j) = \left\{ {\begin{array}{*{20}l} { - {\mathbf{W}}(i,j)} & {i = j + 2} \\ 0 & {i \ne j + 2} \\ \end{array} } \right.$$

where ${\mathbf{W}}(i,j)$ is the $(i,j)$ element of matrix **W**.

Matrix ${\mathbf{\Delta W}}_{Dale}$ was defined as:

$${\mathbf{\Delta W}}_{Dale} (i,j) = \left\{ {\begin{array}{*{20}l} {\;\;\;\;0\;\;\;\;\;} & {({\mathcal{T}}_{j} = Ex\; \wedge \;w(i,j) > 0) \vee ({\mathcal{T}}_{j} = Inh\; \wedge \;w(i,j) < 0)} \\ { - {\mathbf{W}}_{{c_{1} }} (i,j)\;} & {({\mathcal{T}}_{j} = Ex\; \wedge \;w(i,j) < 0) \vee ({\mathcal{T}}_{j} = Inh\; \wedge \;w(i,j) > 0)} \\ \end{array} } \right.$$

where ${\mathbf{W}}_{{c_{1} }} = {\mathbf{W}} + {\mathbf{\Delta W}}_{self}$ and ${\mathcal{T}}_{j} \in \{ Ex,In\}$ indicates if neuron *j* was chosen to be excitatory (*Ex*) or inhibitory (*Inh*). Matrix ${\mathbf{\Delta W}}_{Dale}$ sets to zero the synaptic weights that violate Dale’s principle. Neuron *j* was chosen to be excitatory if $\eta_{j} = \sum\limits_{j} {{\mathbf{W}}_{{c_{1} }} (i,j)} > 0$. Otherwise, it was chosen to be inhibitory. If more excitatory/inhibitory neurons were required, neurons with negative/positive $\eta$ closest to 0 were set as excitatory/inhibitory as needed.

Matrix ${\mathbf{\Delta W}}_{sp}$ to enforce sparsity was defined as:

$${\mathbf{\Delta W}}_{sp} (i,j) = \left\{ {\begin{array}{*{20}l} { - {\mathbf{W}}_{{c_{2} }} (i,j)} & {\left| {{\mathbf{W}}_{{c_{2} }} (i,j)} \right| < \alpha (sp)} \\ 0 & {otherwise} \\ \end{array} } \right.$$

where ${\mathbf{W}}_{{c_{2} }} = {\mathbf{W}}_{{c_{1} }} + {\mathbf{\Delta W}}_{Dale}$. The value $\alpha (sp)$ is the *sp*-percentile of the absolute values in ${\mathbf{W}}_{{c_{2} }}$. In this manner ${\mathbf{\Delta W}}_{sp}$ will set to zero the weights with the lowest absolute value, such that a sparsity *sp* is enforced.

The loss function ${\mathcal{L}}$ at iteration *k* was defined as the average of the absolute ${\mathbf{\Delta W}}_{d} (i,j)$ values:

$${\mathcal{L}}(k) = \left\langle {\left| {{\mathbf{\Delta W}}_{d} (i,j)} \right|} \right\rangle_{i,j}$$

where $\left\langle {} \right\rangle_{i,j}$ stands for the average across indexes $(i,j)$. The structural constraints were imposed through an iterative process, in which neurons were classified as excitatory or inhibitory at each iteration according to their $\eta$, a matrix ${\mathbf{\Delta W}}$ was computed using Eq. (11), and a new **W** was obtained. The process was stopped when the loss fell below a desired value $e_{1}$, in which case the fitting process was considered successful. The process was also stopped if $({\mathcal{L}}(k) - {\mathcal{L}}(k - 1))/{\mathcal{L}}(k) < e_{2}$. When this latter condition was met, the fitting process was considered unsuccessful, since the error was not decreasing fast enough and would probably converge to an unacceptable value above zero. We used $e_{1} = 10^{ - 3}$ and $e_{2} = 10^{ - 4}$. If the process was successful, values that violated any of the constraints were set to zero. These values were expected to be small enough since the error was small. We computed:

$$e_{clip} = \left\langle {\left| {{\mathbf{U}}_{sc} (i,j) - {\mathbf{U}}(i,j)} \right|} \right\rangle_{i,j}$$

where ${\mathbf{U}}_{sc} = {\mathbf{CW}}_{sc}$, with ${\mathbf{W}}_{sc}$ being the resulting synaptic weight matrix after the constraining process, to verify that the deviation from the original **U** was negligible. If the process was unsuccessful, or the clipping error $e_{clip} > 10^{ - 3}$, then the original **W** was considered not to be suitable for the structural fitting.

We measured the efficiency of the process by computing the number of networks generated (*# attempts*) and the running time *t* expended until reaching the first successfully constrained network. We varied $\tau$ from $\tau = 3$ to $\tau = 6$. For each $\tau$ we varied the number of integration neurons in steps of 16 neurons, from a minimum number of $4.2^{\tau }$ to the maximum value $2^{6}$. For each combination of $\tau$ and neuron number we generated networks with the FTP algorithm, and subjected them to structural constraining (no self-connections, 4:1 Ex:In ratio, and a minimum sparsity $sp = 40\%$). We obtained 10 measurements of *# attempts* and *t*, from which we computed the mean and SD depicted in Fig. 4e,f.

### Network construction from random transition graphs

To construct random transition graphs that have an associated consistent system, we first defined a row vector ${{\varvec{\Delta}}}$ and a matrix ${\mathbf{U}}_{lc}$ composed of $M = 2^{\tau }$ row vectors **u** such that ${\mathbf{u}}_{i + 1} - {\mathbf{u}}_{i} = {{\varvec{\Delta}}}$, where **u**_*i*_ is the $i^{th}$ row. Here, indexes *i* are odd numbers between 1 and $M - 1$. Next, we constructed matrix **U** in a way that ensures that each node in the graph was reachable, meaning that every node had to receive at least one edge. This is equivalent to say that every row in **U**_*lc*_ is found at least once in **U**. Therefore, we set the first *M* rows in **U** equal to matrix **U**_*lc*_. The remaining rows in **U** were taken from **U**_*lc*_, picking $M/2$ pairs of indexes $i,i + 1$, choosing *i* values at random from the set of odd numbers between 1 and $M - 1$. In this way, and unlike the transition graphs that solve an s-task, nodes could receive just one edge, or more than two.

So far, if a row vector appeared in matrix **U** more than once, then it appeared only in odd rows, or only in even rows, but not in both. This is because indexes were ordered from 1 to *M* in the first half of **U**, and ordered in pairs of $i,i + 1$ indexes in the second half. The resulting graph would be one in which any given node is reachable as the result of the presentation of either *s*_1_ or *s*_2_, but not from both. In other words, if node *b* is reachable from node *a* after *s*_*i*_ presentation, then node *b* is reachable from node *c* only after *s*_*i*_ presentation, where *c* is any other node from which *b* is reachable. In terms of the colour code of the graph in Fig. 1b, any node receives arrows of the same colour. We wanted graphs as random as possible, so nodes reachable through different stimuli were desired. We defined these nodes as *bicoloured* nodes. In terms of indexes in matrix **U**, a bicolored node translates into a row vector **u**_*i*_ that appeared in matrix **U** in both odd and even rows. For example, if we had indexes $(1,2,3,4)$ for the first 4 rows of **U**, with $({\mathbf{u}}_{1} ,{\mathbf{u}}_{2} ,{{\varvec{\Delta}}})$ and $({\mathbf{u}}_{3} ,{\mathbf{u}}_{4} ,{{\varvec{\Delta}}})$ each being linearly combined, then we wanted to change this series to $(1,2,2,4)$, or $(1,2,3,1)$. This requires to generate new linear combinations, in particular, $({\mathbf{u}}_{1} ,{\mathbf{u}}_{2} ,{\mathbf{u}}_{4} ,{{\varvec{\Delta}}})$ should be linearly combined, for the first example, and $({\mathbf{u}}_{1} ,{\mathbf{u}}_{2} ,{\mathbf{u}}_{3} ,{{\varvec{\Delta}}})$ in the second example. Thus, we modified matrix **U** to generate $f_{bc} M/4$ bicolored nodes, where *f*_*bc*_ stands for ‘bicolored fraction’ and is a number between 0 and 1. The maximum number of bicolored nodes is *M*/4, since we generated one node for each series of indexes *i* to $i + 3$. Finally, we constructed matrix **Z** by thresholding matrix **U**_*lc*_, and then matrix **C**, which rows were in the form:

$$\left( {\begin{array}{*{20}l} {{\mathbf{y}}_{1} {\mathbf{z}}_{k(1)} } \\ {{\mathbf{y}}_{2} {\mathbf{z}}_{k(1)} } \\ \phantom{0,1} \vdots \\ {{\mathbf{y}}_{1} {\mathbf{z}}_{k(M)} } \\ {{\mathbf{y}}_{2} {\mathbf{z}}_{k(M)} } \\ \end{array} } \right)$$

where **z**_*i*_ is the $i{\mathrm{th}}$ row vector in matrix **Z**, and vector *k* is a permutation of the list of integers from 1 to *M*.

Following the above procedure, we constructed random transition graphs that satisfied the linear combinations required so that a consistent system of equations could be constructed. Given that these networks do not solve the s-task but are the minimum Frobenius norm solution to a random transition graph, we call them *Frobenius* (*F)* networks. The procedure avoids index sequences like $(1221)$, since this ordering gives a consistent system only if ${{\varvec{\Delta}}} = {\mathbf{0}}$, in which case stimuli cannot be distinguished by the network. The procedure also avoids index sequences of the type (11) (one node leads to another node through both stimuli, *s*_1_ and *s*_2_). If this were the case, one possibility is that *s*_1_ and *s*_2_ produce the same *u* values (*u* being an element of vector **u**). Therefore, ${{\varvec{\Delta}}}$ is a vector of zeros and stimuli cannot be discriminated. Another possibility is that stimuli lead to different vectors **u**, but these vectors in turn lead to the same vector **z** after thresholding. This situation is possible, but would require careful selection of *u* values in relation to $\theta$, and for this it was avoided.

We constructed networks that followed random transition graphs and compared their properties with the properties of networks that solved the s-task. In particular, we measured the reciprocity of the network, defined as the Spearman correlation between weights of incoming and outgoing synapses. Reciprocity was computed over matrix **W**^*norm*^, a normalized version of the synaptic weights constructed by taking the absolute values of **W** and scaling them between 0 and 1. Since imposing structural constraints like Dale’s principle, or sparsity, may generate many zero-valued weights, reciprocity was computed for synaptic weight pairs such that both $w_{ij}^{norm}$ and $w_{ji}^{norm}$ were both non zero.

For each network generated to solve the s-task we also picked a network from its isofunction space, that is, from the set of all networks that had the same stimulus–response mapping (as described above). We refer to these networks as *Task* (*T*) networks, since they solve the s-task but they are not the minimum Frobenius norm solution. The linear mapping ${\mathbf{\mathcal{M}}}$ in Eq. (10) has entries ${\mathcal{M}}(i,j) = r_{i,j} \mathop {\max }\limits_{i,j} {\mkern 1mu} \left( {{\mathbf{W}}(i,j)} \right)$, where $r_{i,j}$ is a random number, different for each entry, sampled uniformly from the $[ - 1,1]$ interval, and **W** is the synaptic weight matrix from which an isofunction network is desired. We defined mapping ${\mathbf{\mathcal{M}}}$ in this way to obtain isofunction networks with synaptic weight values within the range of the weights in the original network.

In addition, for each network that solves the s-task we constructed an isofunction network with structural constraints: no self-connections and Dale’s principle with excitatory and inhibitory neurons in equal numbers.
